# The CXCL1-CXCR2 Axis Mediates Tubular Injury in Diabetic Nephropathy Through the Regulation of the Inflammatory Response

**DOI:** 10.3389/fphys.2021.782677

**Published:** 2021-12-16

**Authors:** Hanfen Tang, Ming Yang, Yinghong Liu, Hong Liu, Lin Sun, Panai Song

**Affiliations:** ^1^Department of Nephrology, Second Xiangya Hospital, Central South University, Changsha, China; ^2^Department of Nutrition, Second Xiangya Hospital, Central South University, Changsha, China; ^3^Key Laboratory of Kidney Disease and Blood Purification in Hunan Province, Institute of Nephrology, Central South University, Changsha, China

**Keywords:** CXCL1/CXCR2 axis, inflammation, NF-κB, diabetic nephropathy, tubular injury

## Abstract

Diabetic nephropathy (DN) is one of the most severe complications of diabetes. Inflammation mediated by inflammatory factors is thought to accelerate the progression of renal damage in DN. However, which inflammatory factors mediate the inflammatory response in DN remains unclear. In this study, we determined that the CXCL1-mediated inflammatory response may play an essential role in DN progression through bioassays. Subsequently, we observed that the expression of CXCL1 and its receptor (CXCR2) was significantly increased in the kidneys of mice with HFD + STZ induced diabetes and DN patients. In addition, inhibition of the CXCL1/CXCR2 axis by repertaxin alleviates renal inflammation and pathological damage in the kidneys of db/db mice. Finally, we noted that the CXCL1/CXCR2 axis might lead to inflammatory damage through phosphorylated NF-κB and further activate the NLRP3 inflammasome. Our results revealed the role of the CXCL1/CXCR2 axis in DN progression for the first time, which may be a novel therapeutic target for DN.

## Introduction

With the development of the social economy, the number of diabetes patients worldwide is increasing year by year. Prolonged high blood glucose levels can lead to a range of microvascular complications, such as diabetic retinopathy (DR) (Cheung et al., 2010) and diabetic nephropathy (DN) (Yang et al., 2021a). DN has gradually become one of the leading causes of end-stage renal disease (ESRD), which has imposed severe economic burdens on society. Unfortunately, the pathogenesis of DN has not been fully elaborated, and few drugs are available for DN treatment. Mitochondrial dysfunction (Yang et al., 2021b), oxidative stress (Jiang et al., 2020), and other factors are thought to be involved in DN development. Recent studies suggest that immune inflammation may be one of the core factors causing DN (Zheng and Zheng, 2016: Moreno et al., 2018; Yang et al., 2021a).

Multiple inflammatory factors have been reported to be activated in the kidneys of DN mice or patients. El-Horany et al. (2017) demonstrated that the mRNA levels of NLRP3 and IL-1β were increased in DN patients and were positively correlated with the urinary albumin/creatinine ratio and serum creatinine. The inflammatory cytokines MCP-1 (Du et al., 2021), IL-10 (Landis et al., 2018), and IL-6 (Qin and Qiu, 2019) are also increased in the kidneys of DN patients. These studies suggest that inflammation is activated and involved in DN progression. CXCL1 is one of the leading chemical attractants for neutrophils and belongs to the C-X-C chemokine family. It contains a Glu-Leu-Arg (ELR) motif at its amino-terminus (Bischoff et al., 2008), and CXCR2 is its receptor (Yamashiro et al., 2019). Studies have shown that CXCL1 plays a vital role in the development and metastasis of cancers (Acharyya et al., 2012) and an essential role in wound repair and inflammation (De Filippo et al., 2013). In kidney diseases, Liu et al. (2020) demonstrated that CXCL1/CXCR2 mediates the inflammatory response to cisplatin-induced acute kidney injury (AKI). Liu et al. (2020) also reported the role of CXCR2 in ulcerative colitis and Crohn’s disease induced AKI. This evidence suggests that CXCL1/CXCR2 may be involved in renal inflammatory disease. Unfortunately, the role of the CXCL1/CXCR2 axis in DN has rarely been reported.

In the current study, we found that CXCL1 was upregulated in the kidneys of DN patients and db/db mice through GEO databases. Furthermore, we noted that CXCL1/CXCR2 axis and inflammation were activated in HFD-STZ-induced DN mice and DN patients. Additionally, chemical inhibition of the CXCL1/CXCR2 axis relieved renal inflammation, apoptosis, and pathological damage in db/db mice. Finally, we demonstrated that inhibition of the CXCL1/CXCR2 axis might play an anti-inflammatory role by inhibiting the NF-κB/NLRP3 signaling pathway in an *in vitro* study. Our results suggest that the CXCL1/CXCR2 axis is activated in DN and that inhibiting its activation alleviates DN development.

## Materials and Methods

### Microarray Data

Two gene expression datasets (GSE30529 and GSE86300) were obtained from the GEO database, a public functional genomics data repository of high throughput gene expression data.^1^ The GSE30529 dataset contained 10 human diabetic kidney disease renal tissue samples and 12 control tissue samples. The GSE86300 dataset contained 5 db/db mouse renal tissue samples and 5 control tissue samples.

### Animal Experimental Design

Eight-week-old db/db mice obtained from Junke Biological Company (Nanjing, China) were randomly divided into two groups. For repertaxin experiments (db/db + repertaxin, *n* = 6), the mice in the db/db + repertaxin group were treated with repertaxin (20 mg/kg/day) by intragastric administration once every 2 days for 12 weeks. At the age of 20 weeks, the mice were euthanized and kidney, urine and serum samples were harvested for subsequent experimental measurements. To generate HFD + STZ-induced DN mice, 8-week-old C57BL/6 mice were fed a high-fat diet for 4 weeks and then treated with streptozotocin (STZ) (Sigma-Aldrich) in citrate buffer, pH 4.5 (50 mg/kg) for five consecutive days by intraperitoneal injection. After 2 days, the blood glucose level was higher than 16.7 mmol/L, indicating successful modeling of diabetes. After continued feeding for 12 weeks, the mice were euthanized, and kidney and serum samples were harvested for the subsequent experimental measurements. All mice were housed in quiet rooms at 22–26°C with a 12-h light-dark cycle. All animal experiments were conducted in accordance with the relevant institutional guidelines of the Animal Experimentation Ethics Committee of Second Xiangya Hospital of Central South University.

### Human Kidney

Diabetic nephropathy patients (*n* = 6) were diagnosed by renal biopsy and cohorts with non-diabetic renal diseases, such as a minor glomerular lesion (GML) (*n* = 6), were used as the control group. The Institutional Human Experimentation Ethics Committee, Second Xiangya Hospital, Central South University approved and supervised all experiments described above.

### Histological Staining

Hematoxylin-eosin (H&E) and Masson trichrome staining were used to assess kidney damage in 4-μm-thick paraffin-embedded kidney sections from patients with renal biopsy or DN mice. The degree of tubulointerstitial injury was evaluated by a semiquantitative scoring system as described previously (Yang et al., 2019).

### Immunohistochemical Staining

To observe CXCL1/CXCR2 expression and inflammatory infiltration, IHC staining was performed on 4-μm-thick paraffin-embedded kidney sections with a Servicebio immunohistochemical kit (G1212-200T). After deparaffinization, rehydration, antigen repair, and blocking, paraffin sections of renal tissues were incubated with CXCL1 (Proteintech, 1:200), CXCR2 (Proteintech, 1:100), and F4/80 (Servicebio, 1:1,000) for 12 h in 4°C, The tissues were incubated with HRP-conjugated secondary antibody (Abcam, 1:200) for 30min at 37°C. Then, a DAB kit was used for development, followed by observation under a light microscope.

### Analysis of Renal Apoptosis

According to the manufacturer’s instructions (16), terminal deoxynucleotidyl transferase dUTP nick end-labeling (TUNEL) staining was used to evaluate apoptotic cells in renal tissues from different groups (Yang et al., 2019).

### Cell Culture

The human proximal tubular epithelial cell line HK-2 was cultured with DMEM/F12 containing 10% FBS in a 37°C incubator containing 5% CO_2_. After overnight culture with DMEM/F12 without FBS, the HK-2 cells were pretreated with repertaxin (10 μM) 30 min in advance before high-glucose (HG) treatment. After an additional 24 h of culture, the cells were collected for subsequent experiments.

### Quantitative Real-Time PCR

Total RNA was extracted from kidneys and HK-2 cells using TRIzol reagent (Invitrogen) according to the manufacturer’s protocol and quantified by a NanoDrop 2,000 (Thermo Fisher Scientific, Madison, WI, United States). A PrimeScript reagent kit with gDNA Eraser (Takara) was used to synthesize first-strand cDNA. Real-time PCRs were performed with SYBR GreenER qPCR SuperMix (Thermo Fisher Scientific) in a 7300 Real-Time PCR System (Applied Biosystems, CA).

### Western Blot Analysis

Protein from mice and HK-2 cells was collected by radioimmunoprecipitation assay (RIPA) buffer with protease or phosphatase inhibitor, and the protein was quantified by a BCA Protein Assay Kit (Beyotime Biotechnology, China). Then, 5 × SDS loading buffer was added, followed by boiling for 10 min. Equal amounts of proteins were used for western blot analysis.

### Statistical Analysis

SPSS 13.0 software was used for statistical analysis of the experimental data. The results are presented as the mean ± SE. One-way analysis of variance (ANOVA) with Tukey’s *post hoc* analysis was used to compare differences among the groups. Statistical significance was indicated at a *P*-value less than 0.05.

## Results

### CXCL1 May Be an Essential Protein in the Development of Diabetic Nephropathy According to Bioinformatics Analysis

**FIGURE 1:**
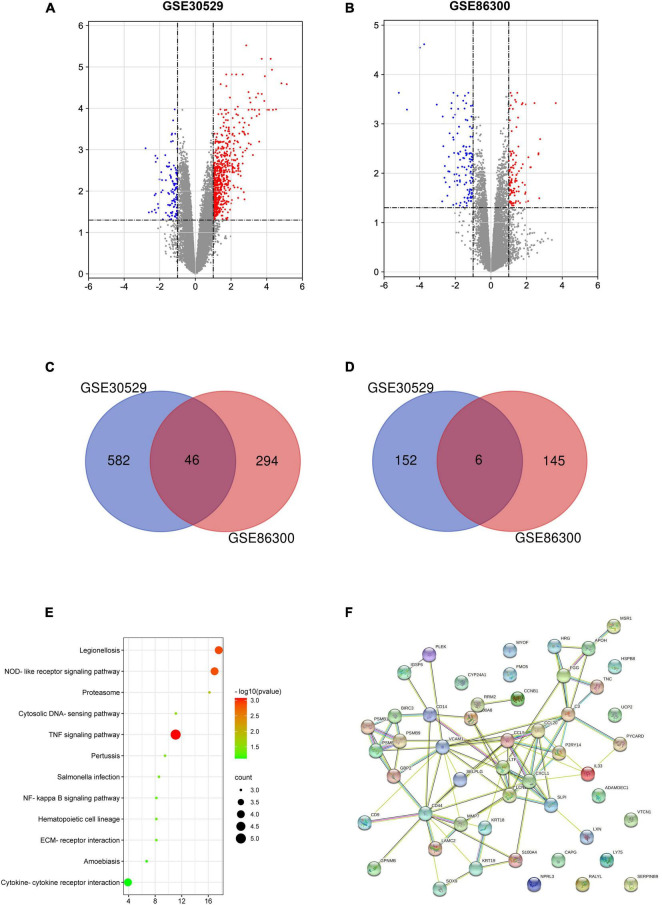
Volcano plot, Venn diagram, PPI network and the most significant module of DEGs. DEGs with a fold change > 1 among the mRNA expression profiling sets GSE30529 **(A)** and GSE86300 **(B)** were selected. Forty-six overlapping of upregulated genes **(C)** and 6 overlapping downregulated genes **(D)** were identified in the two datasets. **(E)** The overlapping genes were analyzed by KEGG analysis. **(F)** The PPI network of overlapping genes.

Through the GEO database, we found that 628 and 340 genes were upregulated and 158 and 151 genes were downregulated, with significant changes in the GSE30529 and GSE86300 datasets, respectively (Figures 1A,B). Forty-six overlapping upregulated genes (Figure 1C) and 6 overlapping of downregulated genes (Figure 1D) were identified in the two datasets. KEGG pathway analysis revealed that these genes were primarily concentrated in the inflammatory pathway (Figure 1E). The PPI network of the 52 DEGs was constructed by the STRING database (Figure 1F).

### Increased Inflammation and CXCL1/CXCR2 Expression Were Observed in the Kidneys of STZ-Induced Diabetic Mice and Diabetic Nephropathy Patients

**FIGURE 2:**
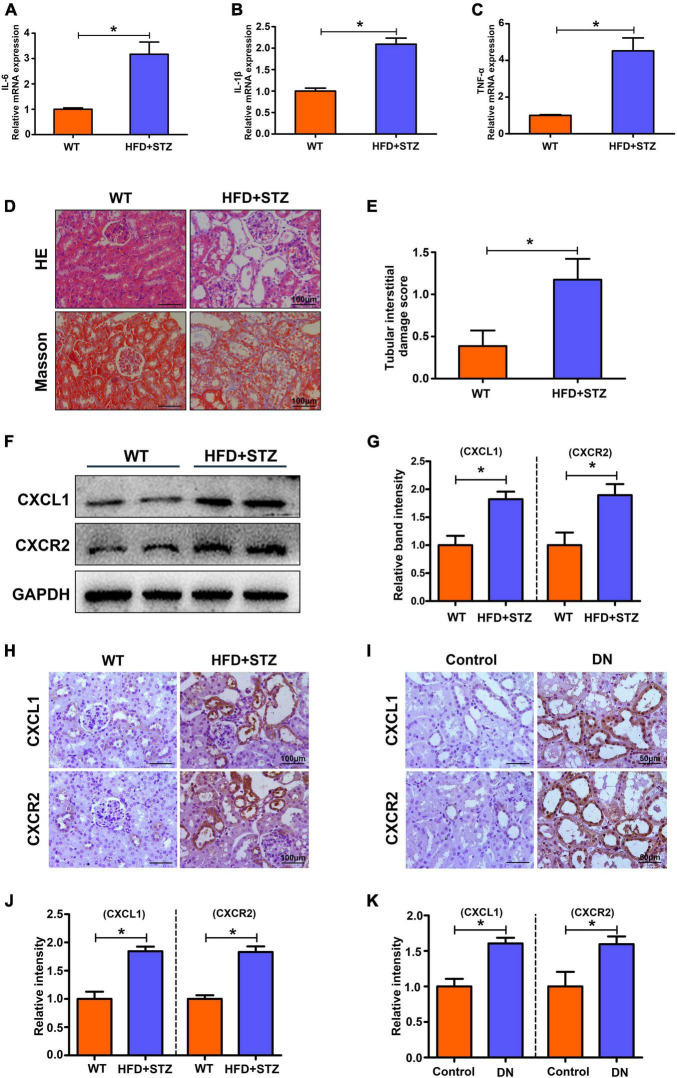
Increased inflammation levels and upregulated CXCL1 or CXCR2 expression in the kidneys of HFD + STZ-induced DN mice and patients. The mRNA levels of IL-6 **(A)**, IL-1β **(B)** and TNF-α **(C)** in mouse kidneys. **(D)** HE and Masson staining to detect pathological changes in the kidneys of WT and HFD + STZ mice. **(E)** Tubular interstitial damage scores of the kidney. **(F,G)** CXCL1 and CXCR2 expression was detected by WB analysis. **(H,J)** IHC staining was used to detect CXCL1 and CXCR2 expression in the kidneys of WT and HFD + STZ mice. **(I,K)** IHC staining was used to detect CXCL1 and CXCR2 expression in renal tissue from patients with renal biopsy. Magnification = 200×. Values are the mean ± SD. Mice *n* = 6/group, patients *n* = 6/group. *p < 0.05 compared with the control group.

To elucidate whether inflammation levels increase in DN states, we detected inflammatory markers in the renal tissues of mice by RT-PCR. As shown in Figures 2A–C, IL-6, IL-1β, and TNF-α mRNA expression was significantly increased in the kidneys of STZ-induced DN mice compared with the control mice. The results show an increased level of inflammation in the kidneys in the DN state. In addition, we investigated the potential role of CXCL1/CXCR2 in the development of DN. HE and Masson staining were used to evaluate pathological lesion changes in the kidneys of DN mice and patients. Exfoliated tubule cells, enlarged lumens, increased fibrosis and increased tubular interstitial damage scores were observed (Figures 2D,E), which were accompanied by increased expression of CXCL1/CXCR2 in DN mice compared to the controls (Figures 2F,G). Similarly, IHC staining also showed increased expression of CXCL1/CXCR2 in DN mice and patients (Figures 2H–K). The results implied that CXCL1/CXCR2 mediated inflammation may play a vital role in the occurrence and development of diabetic nephropathy.

### Repertaxin Inhibits Inflammation and Ameliorates Renal Damage in the Kidneys of db/db Mice

**FIGURE 3:**
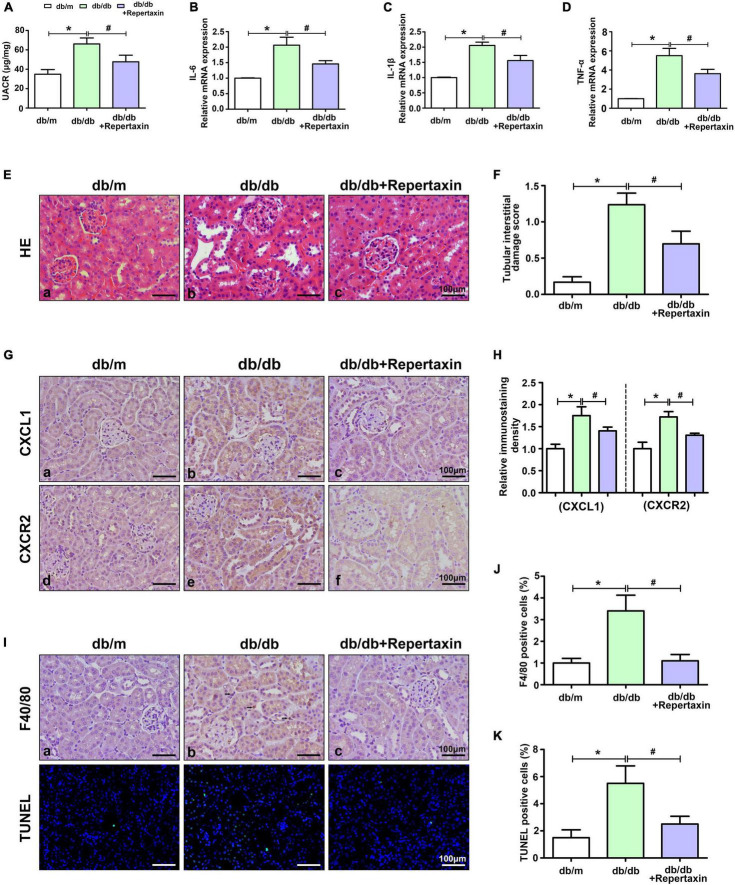
Inhibition of CXCL1/CXCR2 axis activity alleviates renal inflammation and pathological damage. UACR levels **(A)** and the mRNA levels of IL-6 **(B)**, IL-1β **(C)** and TNF-α **(D)** in mouse kidneys. **(E)** HE staining to detect pathological changes in the kidneys of mice. **(F)** The degree of tubular injury was assessed by the tubular interstitial damage score. **(G)** IHC staining was used to detect CXCL1 and CXCR2 expression in kidneys in different groups. **(H)** Histogram representing the levels of CXCL1 and CXCR2. **(I)** F4/80 staining was used to detect inflammatory cell infiltration, and apoptotic cells were detected by TUNEL staining. Histogram representing Inflammatory cell levels **(J)** and apoptotic cell levels **(K)**. Scale bar = 100 μm. The values are the mean ± SD. *n* = 6/group. *p < 0.05 compared with the control group; ^#^*p* < 0.05 compared with the db/db mice.

To further explore the role of the CXCL1/CXCR2 axis in DN development, repertaxin (a previously reported CXCR2 inhibitor) was used to block the CXCL1/CXCR2 axis in db/db mice. Compared with that in the control group, the UACR level was increased in db/db mice and decreased after repertaxin treatment (Figure 3A). Similarly, the mRNA expression of inflammatory makers (IL-6, IL-1β, and TNF-α) (Figures 3B–D) and pathological renal injury (Figures 3E,F) were significantly increased in the kidneys of db/db mice compared with the control group mice, while repertaxin mitigated these adverse changes (Figures 3B–F). Similarly, IHC staining revealed that CXCL1 and CXCR2 expressions were increased in the kidney of db/db mice compared to the db/m group, and their expressions were notably decreased after treatment with repertaxin (Figures 3G,H). F4/80 and TUNEL staining showed increased inflammatory cell infiltration (Figures 3I,J) and apoptosis (Figures 3I,K) in the db/db mice, and repertaxin partially block this effect (Figures 3J,K). These data suggest that when renal tissue is exposed to HG stimulation, the CXCL1/CXCR2 axis is activated, resulting in renal inflammation, while inhibiting the CXCL1/CXCR2 axis with repertaxin may relieve renal inflammation and pathological damage in DN.

### The CXCL1-CXCR2 Axis Mediates the Inflammatory Response Through the NF-κB-NLRP3 Pathway

**FIGURE 4:**
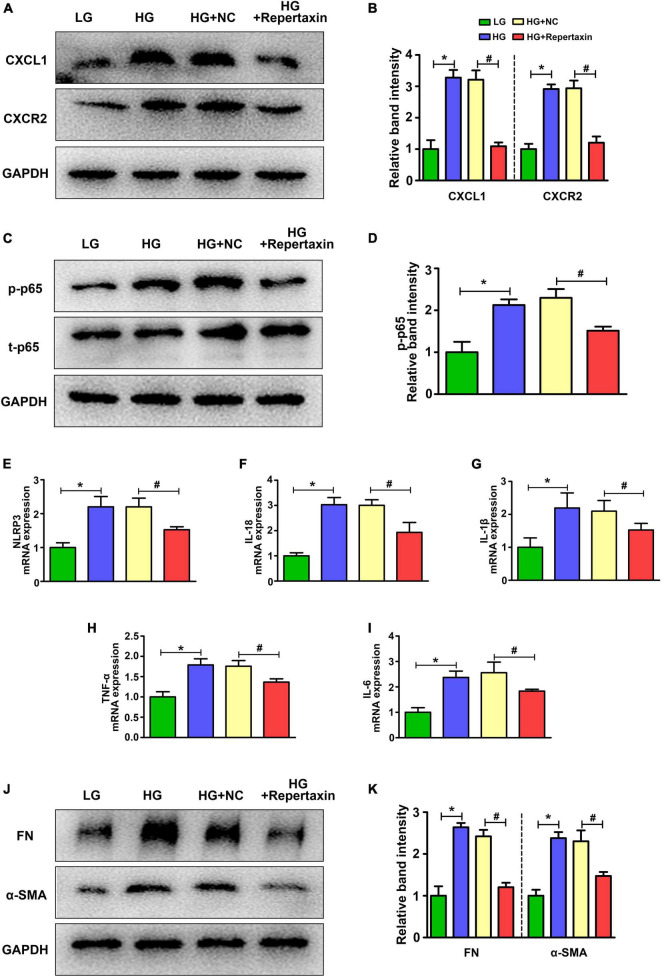
Altered CXCL1/CXCR2 expression and the activated inflammatory response in HK-2 cells treated with HG. **(A,B)** The expression of CXCL1 and CXCR2 in HK-2 cells with different interventions. **(C,D)** Changes in phosphorylated p65 levels in HK-2 cells with different interventions. The mRNA levels of NLRP3 **(E)**, IL-18 **(F)**, IL-1β **(G)**, TNF-α **(H)** and IL-6 **(I)** in HK-2 cells. **(J,K)** The expression of FN and α-SMA in HK-2 cells with different interventions. The values are the mean ± SD. *n* = 6/group. *p < 0.05 compared with the LG group; ^#^*p* < 0.05 compared with the HG + NC group. LG, low glucose; HG, high glucose; HG + NC, high glucose and DMSO; FN, fibronectin; α-SMA, α-smooth muscle actin.

Previous studies have demonstrated that the NF-κB/NLRP3 inflammasome signaling pathway is activated in DN and accelerates renal damage (Yi et al., 2017; Tian et al., 2021), while Liu et al. (2020) also showed that NF-κB is downstream of the CXCL1-CXCR2 signaling pathway. Therefore, we detected the expression of CXCL1/CXCR2-NF-κB-NLRP3 in HK-2 cells with HG intervention. Consistent with the *in vivo* studies, CXCL1/CXCR2 expression was increased in the HG group compared with the LG group, while repertaxin inhibited their expression (Figures 4A,B). Next, we detected the expression of the components in the NF-κB/NLRP3 pathway. Consistent with the changes in CXCL1/CXCR2, the phosphorylation of NF-κB was notably increased in HK-2 cells with HG treatment compared with that in LG group, and when CXCL1/CXCR2 was inhibited, its phosphorylation decreased (Figures 4C,D). In addition, the expression of the NLRP3 inflammasome and other inflammatory factors was detected by RT-PCR. Treatment with HG resulted in an increase in NLRP3 (Figure 4E), IL-18 (Figure 4F), IL-1β (Figure 4G), TNF-α (Figure 4H), and IL-6 (Figure 4I), while suppression of CXCL1/CXCR2 mitigated these adverse changes. Finally, the fibrosis-associated proteins FN and α-SMA were detected by WB analysis. As shown in Figures 4J,K, inhibition of CXCL1/CXCR2 alleviated the increase in fibrosis caused by HG treatment.

## Discussion and Conclusion

Kidney damage caused by diabetes often leads to severe inflammatory responses mediated by activating multiple signaling pathways. The cause of inflammation in DN is unknown but may be associated with a combination of pathogens or tissue damage. However, the former is unlikely to be the most vital contributor, as the kidneys are not usually exposed to pathogens, and no evidence indicates that they are more vulnerable during DN. Improving the understanding of the activation mechanism of the inflammatory response in DN is essential to delay the development of DN. In this study, we noted that inflammatory activation was evident in the kidneys of diabetic mice and DN patients through the GEO database. In addition, we observed that the expression of CXCL1 and CXCR2 was significantly increased in the kidneys of HFD-STZ-induced DN mice and DN patients. Moreover, inhibiting the CXCL1/CXCR2 axis using repertaxin alleviated renal inflammation and pathological damage in db/db mice. Mechanistically, CXCL1/CXCR2 may cause inflammation by phosphorylating NF-κB and activating the NLRP3 inflammasome, and inhibition of the CXCL1/CXCR2 axis can significantly improve the inflammatory response in HK-2 cells with HG treatment.

ROS (Ji et al., 2019), abnormal mitochondrial function (Yang et al., 2017) and other factors are involved in the progression of DN; however, they do not fully explain the pathogenesis of DN, and therefore, no effective clinical drugs are available for the treatment of DN. El-Horany et al. (2017) noted that activation of inflammation was positively correlated with the clinical indicators of DN patients, such as the urinary albumin/creatinine ratio and serum creatinine level. Meanwhile, inflammation was activated in renal biopsy tissues of DN patients or DN mice (Han et al., 2018). This evidence suggests that the role of inflammatory activation in DN cannot be ignored. Here, we noted that the expression of inflammation-related genes was upregulated in the kidneys of diabetic mice and DN patients through the GEO database, and CXCL1 may play an essential role in the activation of inflammation.

As one of the significant chemical attractants of neutrophils, CXCL1 and its receptor CXCR2 play an essential role in the metastasis of cancers, wound repair and inflammation. De Filippo et al. (2013) demonstrated that CXCL1-mediated recruitment of neutrophils is involved in the early stage of tissue inflammation. Moreover, Wang et al. (2017) showed that high CXCL1 expression caused neutrophil infiltration, thus aggravating liver injury, while inhibiting CXCL1 expression ameliorated liver inflammation and damage in steatohepatitis. Similarly, increased expression of CXCL1 and CXCR2 was observed in cisplatin-induced AKI, while chemical agents or gene-knockout inhibition of the CXCL1/CXCR2 axis ameliorated renal injury by inhibiting the inflammatory response (Liu et al., 2020). These findings suggest that activation of the CXCL1/CXCR2 axis is critical in inflammatory disease. Unfortunately, as an inflammation-related disease, the role of CXCL1/CXCR2 in DN has rarely been investigated. This study demonstrated activation of inflammation and upregulation of CXCL1 and CXCR2 expression in the kidneys of HFD-STZ-induced mice and DN patients compared to the controls.

Conversely, inhibiting the CXCL1/CXCR2 axis with repertaxin alleviated renal inflammation and renal injury in db/db mice. In addition, we also observed that HG treatment in HK-2 cells upregulated the phosphorylation of NF-κB and activated the NLRP3 inflammasome, while inhibiting the CXCL1/CXCR2 axis partly blocked these effects, suggesting that the CXCL1/CXCR2 axis may contribute to the downstream inflammatory response by promoting NF-κB and NLRP3 inflammasome activation in DN.

Although we observed this phenomenon and the possible mechanism, future work needs to construct renal tubular cell-specific CXCL1 or CXCR2 knockout mice to verify this pathway *in vivo*. In addition, some key questions also remain to be resolved. For instance, what is the precise molecular mechanism by which CXCL1/CXCR2 inhibits NF-κB and NLRP3 inflammasome activation? Although much work still needs to be carried out, this study provides new insights into the treatment of DN.

## Data Availability Statement

Publicly available datasets were analyzed in this study. This data can be found here: http://www.ncbi.nlm.nih.gov/geo; GSE30529 and GSE86300.

## Ethics Statement

The studies involving human participants were reviewed and approved by Medical Ethics Committee of Central South University. The patients/participants provided their written informed consent to participate in this study. The animal study was reviewed and approved by the Institutional Animal Care and Use Committee (IACUC) of Central South University.

## Author Contributions

HT designed the study, analyzed the data, interpreted the results, and drafted the manuscript. MY, YL, HL, and LS contributed to the data collection and manuscript revision. PS was involved in the study design, data interpretation, and manuscript revision. All authors contributed to the article and approved the submitted version.

## Conflict of Interest

The authors declare that the research was conducted in the absence of any commercial or financial relationships that could be construed as a potential conflict of interest.

## Publisher’s Note

All claims expressed in this article are solely those of the authors and do not necessarily represent those of their affiliated organizations, or those of the publisher, the editors and the reviewers. Any product that may be evaluated in this article, or claim that may be made by its manufacturer, is not guaranteed or endorsed by the publisher.

## Footnotes

1
http://www.ncbi.nlm.nih.gov/geo
